# Criteria of acceptance in the Saudi program of anesthesia and intensive care

**DOI:** 10.4103/1658-354X.57881

**Published:** 2009

**Authors:** Jamal A. A. Tashkandi

**Affiliations:** *Department of Anesthesia, Consultant and Assistant Professor of Anesthesia, College of Medicine, King Khalid University, Abha, Kingdom of Saudi Arabia*

**Keywords:** *Saudi Anesthesia Program*, *acceptance criteria*, *resident training program*

## Abstract

**Background:**

The scientific congress of Anesthesia and Intensive Care of the Saudi Commission for Health Specialties aims to review and improve the guidelines for the selection process of trainees, a selection process that is based on equal opportunity and upholds the principles of consistency, objectiveness, transparency, and procedural fairness. The study represents a step toward the goal of fostering quality patient care, by adopting a selection process that would result in graduating good, committed, and competent specialists.

**Materials and Methods:**

Reports of admission examinations in Jeddah, Riyadh, and the Eastern region have been collected, and they contain detailed lists of names, scores, and percentages of the criteria of admissions, that is, MBBS 25%, General Examination 50%, Interview 25%, and overall score of 100%.

**Results:**

Mean MBBS scores, average general examination scores, average interview scores, and average overall scores were not statistically different between candidates from different regions. The leading predictor was the ‘Interview Score’. 49.5% of variation in the dependent variable (overall score) could be significantly explained (F = 69.4, *P* < 0.05) by the independent variable ‘Interview Score’. The second predictor was the ‘MBBS score’.

**Conclusion:**

The three components MBBS, General Examination, and Interview, were significant predictors of the overall score. The leading predictor was the ‘Interview Score’. The author recommended that the selection process should be under continuous review. The general interview guide approach is recommended to ensure that the same general areas of information are collected from each interviewer. Questions of a personal or discriminatory nature should be avoided.

## INTRODUCTION

The Scientific Congress of Anesthesia and Intensive Care of the Saudi Commission for Health Specialties is aiming to apply for a scientifically evidenced guideline for the selection process of trainees, which can be available on its website[1,2], a selection process that is based on equal opportunity and upholds the principles of consistency, objectiveness, transparency, and procedural fairness. This study represents one of the many steps toward the goal set by the scientific congress of anesthesia and intensive care, which is fostering quality patient care by adopting a selection process, which could result in graduating good, committed, and competent specialists.

## MATERIALS AND METHODS

Reports of admission for the year 2009-2010 of Jeddah, Riyadh, and the Eastern region have been collected from the office of the Scientific Congress of Anesthesia and Intensive Care of the Saudi Health Commission. Each report contains detailed lists of names, scores, and percentages of the three criteria of admissions, as per the guidelines of the Saudi Program of Anesthesia and Intensive Care, which follow the rules of the Saudi Health Commission, that is, MBBS 25%, General Examination 50%, Interview 25%, and an overall score of 100%. Frequency, arithmetic mean, and standard deviation have been used to present the data. Chi square and ANOVA have been used as a test of significance at the 5% level. Regression analysis has been used to study the predictors of overall scores.

## RESULTS

The study included the data of 73 candidates who attended the admission examinations in Jeddah, Riyadh, and the Eastern region. The original cohort included 77 candidates, however, the data were not complete for four candidates. They were omitted from the final analysis. The majority of applicants were from Riyadh (41,56.2%), followed by Jeddah (23, 31.5%), and the Eastern Region (9, 12.3%). The mean MBBS scores among candidates from different regions were not statistically significant from each other (F = 2.162, *P* = 0.123). Similarly other scores (average general examination scores, average interview scores, and average overall scores) were not statistically different between candidates from different regions.

**Figure 1:**
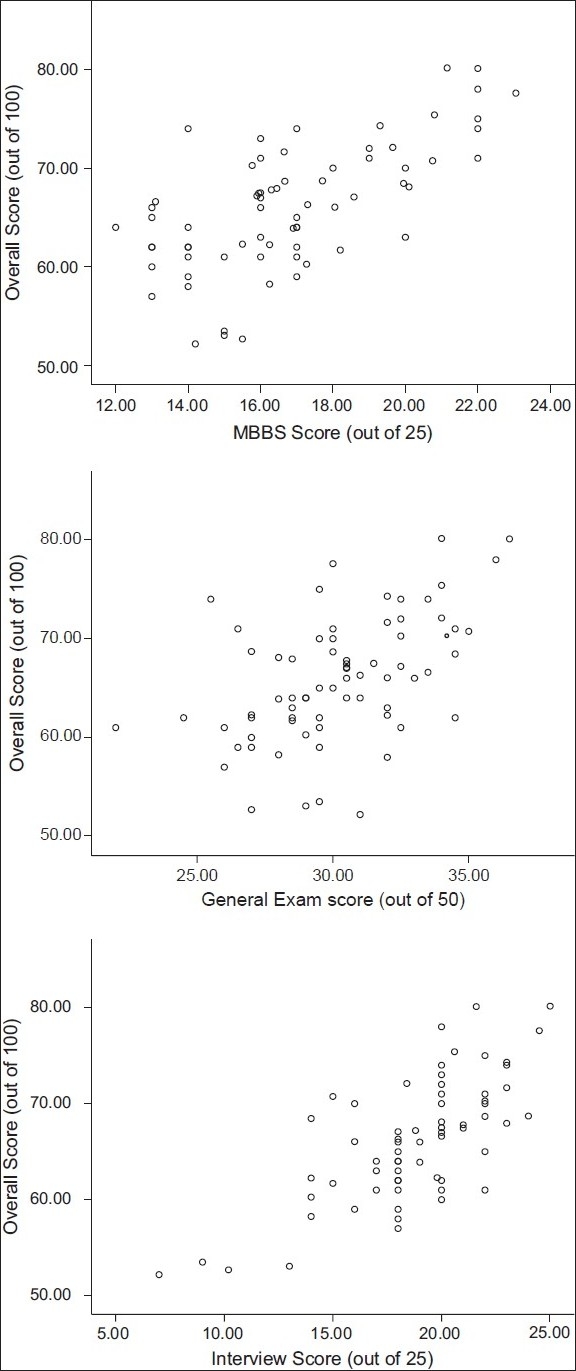
Scatter gram of overall performance and different examination components

Table 1 and Figure 1 show that the three components were significant predictors of the overall score. The leading predictor is the ‘Interview Score’, 49.5% of variation in the dependent variable (overall score) can be significantly explained (F = 69.4, *P* < 0.05) by the independent variable ‘Interview Score’. The second predictor is the ‘MBBS score’, 44.3% of variation in the dependent variable (overall score) can be significantly explained (F = 56.2, *P* < 0.05) by the independent variable ‘MBBS score’.

**Table 1:** Regression analysis: Performance in different examination components and overall score of all candidates

Variables entered	Model summary		ANOVA		Coefficients		
			
	R	R square	F	P	B	t	P
MB BS score	0.665	0.443	56.2	0.001*	1.56	7.586	0.001*
General examination score	0.509	0.259	24.8	0.001*	1.10	4.986	0.001*
Interview score	0.703	0.495	69.4	0.001*	1.32	4.886	0.001*

Significant (*P* < 0.0)

The general examination score ranked third as a predictor of the overall score. Only 25.9% of the variation in the dependant variable ‘Overall score’ could be significantly explained by the independent variable ‘General Examination score’. Table 2 shows that 52.1% (38) of the candidates were accepted. On the other hand, 24.7% of the candidates were rejected. The highest acceptance rate was in Riyadh (58.5%, 24), followed by the Eastern region (44.4%, 4) and Jeddah (43.5%, 10). The difference in distribution was statistically significant (Chi square value = 10.177, *P* = 0.038).

**Table 2:** Final results of the candidates

	Frequency	%
Valid		
Accepted	38	52.1
Waiting	17	23.3
Rejected	18	24.7
Total	73	100.0

## DISCUSSION

This study shows that candidate scores (MBBS, General Examination, Interview, and Overall) are not different from each other by region. The strongest predictor of the overall score is the interview score. The acceptance rate is different by region. Even overall scores among the accepted are different by region. It seems that the acceptance — setting up the cut off point for acceptance — is preset and governed mainly by the number of available places rather than by the performance, which might explain this variation in spite of being similar in everything else as a group. The current selection components of acceptance stated by the Scientific Congress of Anesthesia and Intensive Care of the Saudi Commission for Health Specialties could be divided into two main components, the academic record of achievement of the candidates, which is represented by both the MBBS score and the postgraduate General Examination, which carries 75% of the overall score, and a second component, which is the interview, which carries only 25%. It was not obvious from the gathered data whether the interview panels prepared core questions for the interview. In addition, no specific duration of time for the interview was mentioned in the reports.

Whether or not the interview is a good predictor of acceptance for the selection process of a good, committed, and competent specialist is yet to be decided. Currently this study cannot claim that it can answer such questions; despite the fact that it shows clearly that the strongest predictor of the overall score is the interview score. A further follow-up study could measure the performance of the currently accepted candidates, for example, results of the part I examination of the program could be compared with the findings of the follow-up study, and this could answer the above-mentioned question. The author has recommended that the selection process be under continuous review. Increasing the percentage of the interview score out of the overall score should be considered. The interview panel should have a set of core questions, previously prepared by a selection committee, which should not be the same as those set by the interview panel. Additional questions that may cover areas such as good health and conduct, as well as, appropriate interpersonal and communication skills may be asked, if necessary. Questions of a personal or discriminatory nature, relating to religion, marital status, sexual preferences, and parenthood should be avoided. A general interview guide approach is recommended, as it is intended to ensure that the same general areas of information are collected from each interviewer; this provides more focus than the conversational approach, but still allows a degree of freedom and adaptability in getting information from the interviewer. Adding references for the candidates as a mandatory prerequisite component of the selection process may reflect a professional opinion regarding the personality of the candidates such as acceptance of teamwork, reliability, and responsibility.

## CONCLUSION

The three components MBBS, General Examination, and Interview, were significant predictors of the overall score. The leading predictor was the ‘Interview Score’. The author recommended that the selection process should be under continuous review. A general interview guide approach is recommended to ensure that the same general areas of information are collected from each interviewer. Questions of personal or discriminatory nature should be avoided.
